# Spermidine restores dysregulated autophagy and polyamine synthesis in aged and osteoarthritic chondrocytes via EP300: response to correspondence by Borzì et al.

**DOI:** 10.1038/s12276-019-0225-3

**Published:** 2019-03-01

**Authors:** Pradeep K. Sacitharan, George Bou Gharios, James R. Edwards

**Affiliations:** 1 Botnar Research Centre, University of Oxford, Oxford, UK; 2 The Institute of Ageing and Chronic Disease, University of Liverpool, Liverpool, UK

**Keywords:** Macroautophagy, Mechanisms of disease

We welcome the comments and research efforts by Borzì et al. mirroring our work highlighting the significance of longevity-related mechanisms in skeletal biology (sirtuins, autophagy) and how naturally occurring non-specific products (polyamines, polyphenols) might prolong cell mortality. Specifically, Borzì et al. appear to agree with our recent conclusion that spermidine activates autophagy and is chondroprotective, yet the intricate dynamics of epigenetic and/or post-translational modifications at play within this system have served to obfuscate this message. Borzì et al. correctly indicate that our referenced literature describes known interactions between the EP300 acetyltransferase and a selection of the 170 proteins forming the human autophagy protein network. We intentionally highlight the varied and contradictory effects of protein acetylation upon normal cellular processes, suggesting that fine tuning of autophagy occurs through changes in epigenetic modifiers, and which might alter with disease state or environmental stimuli.

Our data, using primary human chondrocytes, show treatment with spermidine increases EP300 while molecular knockdown with EP300 siRNA abrogates spermidine-induced autophagy. Our use of chondrocytic cells to demonstrate endogenous acetylation of autophagy-related proteins is more relevant to bone and joint disease than the cell-free system described by Pietrocola et al. using recombinant EP300 protein, or exploring EP300-autophagy associations in mutated cancer cell lines^1^. We agree with Borzì et al. and accept findings from studies demonstrating varied interactions between spermidine, EP300 and autophagy in a variety of cell types and experimental conditions; however, we do not believe that this complex system can be reduced to an ‘on/off’ dynamic, where decreased EP300 will always increase protein activity. As indicated by Pietrocola et al., changes in individual post-translational modifiers do not necessarily correlate with alterations in acetylation status or in this case, autophagic flux. It is not simply a state of either ‘inhibition’ or ‘activation’ by a given protein; both are possible depending on the circumstance. In further example, Banreti et al. describe EP300 forming protein complexes with CREBBP/KAT3A and KAT5/TIP60, inducing autophagy through increased acetylation^2,3^. Similarly, increased acetylation by EP300 increases protein activity and might underlie tumour development^4^, pulmonary fibrosis^5^, while decreased EP300-induced acetylation led to a concurrent decrease in glycolytic activity^6^ and contributes to skeletal malformations^7^. Furthermore, the cellular localisation and activity of EP300 upon autophagy-related proteins are further modulated by co-factors and environmental stress (serum starvation)^8^, indicating that EP300-induced acetylation in one cell type is not always predictable and can be modified by external influences. Confounding this system further, spermidine is associated with both hyper- and hypo-acetylation of autophagy-related proteins^9,10^.

It is clear that autophagy is a complex process mediated by numerous proteins and subject to local stimuli and epigenetic modification, complicating the study and analysis of this system. Throughout our work, we have routinely used standard controls in our analysis of autophagic flux, and where the addition of lysosomal protease inhibitors did not provide further relevant information. We value the contribution by Borzì et al. in highlighting the intricacy of this system where timing, external milieu and different regulatory processes converge to manifest as a temporal autophagic response.
